# Longitudinal neuroimaging and neuropsychological profiles of frontotemporal dementia with *C9ORF72 *expansions

**DOI:** 10.1186/alzrt144

**Published:** 2012-09-24

**Authors:** Colin J Mahoney, Laura E Downey, Gerard R Ridgway, Jon Beck, Shona Clegg, Melanie Blair, Sarah Finnegan, Kelvin K Leung, Tom Yeatman, Hannah Golden, Simon Mead, Jonathan D Rohrer, Nick C Fox, Jason D Warren

**Affiliations:** 1 Dementia Research Centre, University College London Institute of Neurology, London WC1N 3BG, UK; 2 Wellcome Trust Centre for Neuroimaging, University College London Institute of Neurology, London WC1N 3BG, UK; 3 MRC Prion Unit, University College London Institute of Neurology, London WC1N 3BG, UK

## Abstract

**Introduction:**

Frontotemporal dementia (FTD) is a common cause of early-onset dementia with a significant genetic component, as underlined by the recent identification of repeat expansions in the gene *C9ORF72 *as a major cause of FTD and motor neuron disease. Understanding the neurobiology and clinical phenomenology of this novel mutation is currently a major research focus. However, few data are available concerning the longitudinal evolution of this genetic disease. Here we present longitudinal neuropsychological and neuroimaging data on a cohort of patients with pathological repeat expansions in *C9ORF72*.

**Methods:**

Following a review of the University College London FTD DNA database, 20 cases were retrospectively identified with a *C9ORF72 *expansion. Twelve cases had longitudinal neuropsychology data available and six of these cases also had longitudinal volumetric brain magnetic resonance imaging. Cortical and subcortical volumes were extracted using FreeSurfer. Rates of whole brain, hemispheric, cerebellar and ventricular change were calculated for each subject. Nonlinear fluid registration of follow-up to baseline scan was performed to visualise longitudinal intra-subject patterns of brain atrophy and ventricular expansion.

**Results:**

Patients had low average verbal and performance IQ at baseline that became impaired (< 5th percentile) at follow-up. In particular, visual memory, naming and dominant parietal skills all showed deterioration. Mean rates of whole brain atrophy (1.4%/year) and ventricular expansion (3.2 ml/year) were substantially greater in patients with the *C9ORF72 *mutation than in healthy controls; atrophy was symmetrical between the cerebral hemispheres within the *C9ORF72 *mutation group. The thalamus and cerebellum showed significant atrophy whereas no cortical areas were preferentially affected. Longitudinal fluid imaging in individual patients demonstrated heterogeneous patterns of progressive volume loss; however, ventricular expansion and cerebellar volume loss were consistent findings.

**Conclusion:**

Disease evolution in *C9ORF72*-associated FTD is linked neuropsychologically with increasing involvement of parietal and amnestic functions, and neuroanatomically with rather diffuse and variable cortical and central atrophy but more consistent involvement of the cerebellum and thalamus. These longitudinal profiles are consistent with disease spread within a distributed subcortical network and demonstrate the feasibility of longitudinal biomarkers for tracking the evolution of the *C9ORF72 *mutation phenotype.

## Introduction

Frontotemporal dementia (FTD) is characterised by early behavioural change and progressive erosion of social cognition associated with frontotemporal lobar degeneration [1]. A substantial number of cases of FTD have a familial basis [2], and an expanded hexanucleotide (GGGGCC) repeat insertion in a noncoding promoter region of ORF 72 of chromosome 9 (*C9ORF72*) was recently identified as an important cause of FTD and motor neuron disease [3,4]. Recent reports of *C9ORF72 *mutations suggest these are a common cause of FTD and motor neuron disease, representing approximately one-third of all cases due to genetic mutations [5,6], of comparable frequency to mutations in progranulin (*GRN*) and micro-tubule protein tau (*MAPT*) as a cause of autosomal dominant FTD [6].

Clinically, *C9ORF72 *expansions have been associated with a behavioural dysexecutive phenotype but also with notable early features including psychosis and anxiety as well as impaired episodic memory [6,7]. Individual cross-sectional magnetic resonance imaging (MRI) has revealed a highly variable imaging phenotype with involvement of frontal, temporal and parietal cortices, and limited previous longitudinal data have suggested similar rates of whole brain atrophy in *C9ORF72 *and *MAPT *mutation cases [6]. Group-level cross-sectional imaging studies have confirmed this distributed pattern of atrophy as well as emphasising additional cerebellar and subcortical involvement [6,8,9]. Both imaging and clinical studies suggest that the neurodegenerative process associated with the *C9ORF72 *expansion is rather diffuse [10]. Whilst early symptoms are most in keeping with frontal lobe dysfunction, parietal dysfunction becomes more apparent as the disease progresses [6]. These clinical features suggest that the disease may propagate along a rostrocaudal gradient, perhaps spreading via a distributed brain network. Understanding the clinical and radiological evolution of *C9ORF72-*associated FTD is an important issue. Detailed studies of longitudinal imaging profiles and neuropsychological changes associated with *C9ORF72 *mutations remain limited: longitudinal studies may enable evaluation of candidate biomarkers both for diagnosis and future clinical trials of disease-modifying agents. More fundamentally, the concept of network-led degeneration is gaining currency as an important general theme in neurodegeneration [11] and *C9ORF72*-associated FTD, as a novel genetic proteinopathy, may offer fresh insights into the mechanisms of neurodegenerative disease propagation.

Here we present longitudinal data on a cohort of patients with FTD associated with *C9ORF72 *expansions. We detail profiles of neuropsychological progression, rates of whole brain, cerebellar and subcortical atrophy and anatomical profiles of disease progression using nonlinear fluid registration of serial MRI.

## Methods

### Case ascertainment

Twenty cases from a previously published DNA cohort comprising 227 cases within the frontotemporal lobar degeneration spectrum [6] were found to harbour a *C9ORF72 *expansion using the repeat-primed PCR as previously published [4]. Mutations were called where more than 30 repeats were shown consistently. For the purpose of reporting longitudinal change only cases with a minimum of two neuropsychological assessments or volumetric MRI scans were included. In total 12 individuals (mean age 59.4 years (± 6.8 years), seven male) had longitudinal neuropsychological data and six of these cases (mean age 62.7 years (± 7 years), five male) also had longitudinal volumetric MRI scans. All cases identified had been assessed in the Specialist Cognitive Disorders Clinic at the National Hospital for Neurology and Neurosurgery by an experienced cognitive neurologist and all met current consensus criteria for a diagnosis of behavioural variant FTD [1]. Two cases had additional clinical features of motor neuron disease at presentation.

The study was approved by the local research ethics committee under Declaration of Helsinki guidelines and all subjects gave informed consent for participation.

### Neuropsychological assessment

The mean duration between serial neuropsychological assessments was 1.4 years (± 0.7 years). General intellectual function was assessed using the Wechsler Adult Intelligence Scale - Revised or the Wechsler Abbreviated Scale of Intelligence [11,12]. Executive function was assessed using the Weigl test, the Stroop colour-word test or the Hayling test [13-15]. Verbal memory and visual memory were assessed with the Recognition Memory Test for words and faces respectively [16]. Naming was assessed using the Graded Naming Test or the Oldfield Naming Test [17,18]. Visuospatial and visual perception skills were assessed using subsets of the Visual Object and Spatial Perception battery [19]. Calculation and spelling were assessed with the Graded Difficulty Arithmetic test [20] and the Baxter Spelling tests [21] respectively. Raw scores were converted into percentiles for reporting.

### Brain image acquisition and processing

Serial T_1_-weighted magnetic resonance volumetric brain MRI was performed using a Magnetization Prepared Rapid Gradient Echo sequence: three studies were acquired on a 1.5T GE Signa scanner (General Electric Milwaukee, WI, USA) (256 × 256 matrix; 1.5 mm slice thickness) and three studies acquired on a 3.0T Siemens Trio scanner (Siemens, Germany) (256 × 256 matrix; 1.1 mm slice thickness). Patient data were compared with data from 15 age-matched (mean age 57.7 years; 10 male, five female) healthy controls with two volumetric MRI scans (12 controls on the 1.5T scanner, three controls on the 3.0T scanner). The mean duration between scans was 1.0 years (± 0.2 years) for patients and 1.6 years (± 0.8 years) for controls. All images were visually inspected for alternative pathologies and motion artefact. Whole brain, ventricular and cerebellar segmentation was performed by an experienced segmentor using a semi-automated technique using the MIDAS software package [22]. Scans underwent affine registration to spatially align the repeat scan to baseline. Rates of whole brain and cerebellar atrophy and ventricular expansion were calculated using the boundary shift integral (BSI), utilising the more robust KN-BSI methodology to provide automatic quantification of volume change[23]. Rates of change are expressed as the percentage loss from baseline volume and adjusted to an annualised rate according to the interval. Scans were registered into standard space for ventricular and hemispheric segmentation. Ventricular regions included the lateral ventricles and temporal horn of the lateral ventricles but excluded the third and fourth ventricles. Visualised cerebellar regions were dissected from the brainstem at mid-pontine level and images underwent further manual editing in coronal and sagittal planes to remove any remaining areas of brainstem. Right and left cerebral hemispheric volumes were calculated by dividing the brain along the mid-sagittal section. Finally, total intracranial volumes were calculated by summing grey matter, white matter and cerebrospinal fluid volumes acquired using the New Segment toolbox within Statistical Parametric Mapping 8 [24,25].

Cortical and subcortical regional volumes were obtained from each subject's baseline and repeat volumetric MRI images using FreeSurfer (v5.1) running the automated longitudinal processing stream [26]. Default parameters were used with the exception of applying custom brain masks defined from the whole brain segmentation step to improve anatomical accuracy. Segmentations were visually inspected and edited where necessary. Volumes from 34 cortical regions following the Desikan atlas [27] and six subcortical regions (thalamus, caudate, putamen, globus pallidus, amygdala, hippocampus) were extracted for each subject and time point.

Following affine registration and bias correction of scan pairs, each scan set underwent cropping using subject-specific masks to exclude nonbrain regions. Fluid registration was performed to visualise intra-subject changes in brain morphology [28]. Briefly this involves nonlinear warping of each individual's repeat scan to match their baseline scan, generating a deformation field for each subject that allows visualisation of voxel-level expansion or contraction.

## Results

### Neuropsychological findings

**Figure 1:**
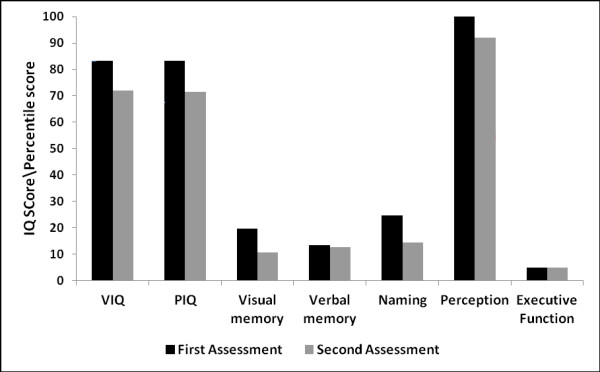
**Group-level longitudinal performance on neuropsychological measures**. Verbal IQ (VIQ) and performance IQ (PIQ) reported as raw scores. For the purpose of standardising across tests and to visualise changes in performance, raw scores were converted to percentiles. Where a score of 50th to 75th percentile was reported this was converted to the median value (that is, 62.5) for visualisation.

Group-level performance is displayed in Figure 1 and individual data in Table 1. At baseline the mean general intellectual function, as reflected in verbal IQ and performance IQ, was in the low average range (mean baseline verbal IQ = 83 (± 14); mean baseline performance IQ = 83 (± 15)); over the period of follow-up, both verbal IQ and performance IQ became impaired, declining by an average of 11 points (mean follow-up verbal IQ = 72 (± 19); mean follow-up performance IQ = 71 (± 23)).

**Table 1:** Individual neuropsychological data for baseline and follow-up assessments

Case	Syndrome	Disease duration (years)	Assessment number	VIQ	PIQ	RMT-F	RMT-W	Naming	VOSP %	Executive function^a^
1	bvFTD	34.5	1	106	113	< 5th	5th to 10th	95th	Pass	< 5th
		4.5	2	110	110	2nd to 10th	N/A	> 25th	Pass	< 5th
2	bvFTD	3	1	87	78	< 5th	< 5th	< 5th	Pass	< 5th
		4.7	2	81	76	25th	< 10th	< 1st	Pass	< 5th
3	bvFTD	7	1	85	69	50th to 75th	10th to 25th	< 5th	Pass	< 5th
		7.7	2	68	71	< 5th	5th to 10th	5th to 10th	Fail	< 5th
4	bvFTD	3	1	73	78	< 5th	< 5th	5th to 10th	Pass	< 5th
		4.5	2	NT	NT	< 5th	< 5th	< 1st	Pass	Reduced
5	bvFTD	11	1	116	113	50th to 75th	75th to 95th	75th	Pass	Pass
		14.4	2	101	114	50th to 75th	> 95th	> 75th	Pass	Pass
6	bvFTD	2.75	1	69	69	< 5th	< 5th	< 5th	Pass	< 5th
		3.75	2	57	67	< 5th	< 5th	5th to 10th	Pass	< 5th
7	bvFTD	1	1	80	89	< 5th	< 5th	75th	Pass	Borderline
		2.8	2	59	63	< 5th	< 5th	< 5th	Pass	< 5th
8	FTD-MND	3	1	69	80	< 5th	5th to 10th	5th to 10th	Pass	< 5th
		4.2	2	67	65	< 5th	< 5th	5th to 10th	Pass	< 5th
9	bvFTD	2	1	78	76	< 5th	< 5th	> 5th	Pass	Borderline
		3	2	78	NT	< 5th	< 5th	> 25th	Pass	< 5th
10	bvFTD	5	1	82	77	Normal	< 5th	< 5th	Pass	Pass
		6.6	2	55	77	< 5th	< 5th	< 1st	Pass	< 5th
11	FTD-MND	1.5	1	84	82	10th	5th to 10th	< 5th	Pass	< 5th
		2.5	2	77	60	< 5th	< 5th	5th to 10th	Pass	< 5th
12	bvFTD	3	1	71	75	< 5th	< 5th	< 5th	Pass	Pass
		3.7	2	71	74	< 5th	< 5th	N/A	Pass	Pass

Raw scores are displayed for verbal IQ (VIQ) and performance IQ (PIQ); otherwise results are displayed as percentiles. Pass, > 5th percentile; borderline, 5th to 10th percentile. bvFTD, behavioural variant frontotemporal dementia; FTD-MND, frontotemporal dementia-motor neuron disease; N/A, not available; NT, not testable; RMT-F, Recognition Memory Test for faces; RMT-W, Recognition Memory Test for words; VOSP, visual object and space perception battery. ^a^Tests of executive function (Weigl test, Stroop colour-word test or Hayling test).

Executive function was severely impaired in the majority of subjects at baseline (7/12 subjects scored < 5th percentile on at least one executive measure) and deficits became more frequent over the period of follow-up (10/12 < 5th percentile). Recognition memory was frequently weak at baseline, with deficits in verbal (7/12 < 5th percentile) and visual memory (8/12 < 5th percentile); over the period of follow-up, visual memory deficits became more frequent (10/12 < 5th percentile). Naming was impaired in one-half of the patients at baseline (6/12 < 5th percentile); at follow-up, naming deficits were evident in the majority (8/12 < 5th percentile).

Dominant parietal skills were assessed in only five patients longitudinally; however, three had evidence of dyscalculia and/or dysgraphia at baseline and four exhibited at least one of these deficits at follow-up. Visual perceptual functions remained largely stable over the period of follow-up, only one subject becoming impaired.

### Neuroimaging findings: atrophy rates

**Figure 2:**
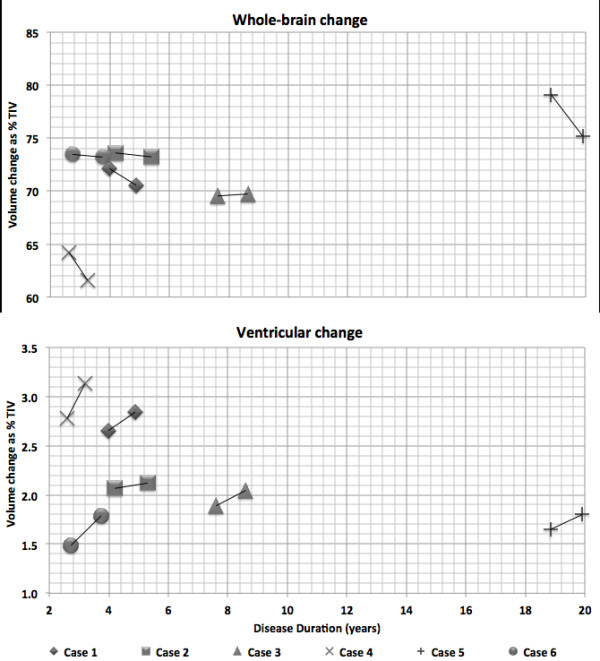
**Change in brain volume and ventricular volume**. Change in brain volume (upper panel) and ventricular volume (lower panel) for each subject expressed as a proportion of the total intracranial volume (TIV) and modelled as a function of disease duration.

Individual and group volumetric data and rates of whole brain, hemispheric and ventricular change measured using BSI are displayed in Figure 2 and Table 2. Rates of whole brain atrophy varied widely between subjects; these data have been reported previously for five of the cases in the present series [6]. The most consistent finding (present in 5/6 cases) was an increased rate of ventricular enlargement in patients with a *C9ORF72 *mutation: patients had a mean annualised ventricular expansion rate of 3.2 (± 2.0) ml/year compared with controls at 0.7 ml/year (± 0.6) (*P *= 0.001), despite substantial individual variation. Longitudinal cerebellar atrophy was present in the majority of individual patients (4/6 cases); the mean annualised rate of cerebellar atrophy in the patients was also significantly higher (1.0%/year) than in controls (0.1%/year; *P *= 0.02). In addition, the mean annualised rate of whole brain atrophy in the patients as measured using KN-BSI (1.4%) was significantly higher than in controls (0.4%; *P *= 0.04) Mean atrophy rates for the cerebral hemispheres considered separately were similar for each hemisphere (left 2.4%/year; right 2.1%/year) and similar to the whole brain atrophy rate; atrophy in individual patients was symmetrical between the hemispheres across the *C9ORF72 *mutation group (inter-hemispheric volume ratio 0.99) and did not become more asymmetric over the follow-up interval.

**Table 2:** Individual patient and group brain magnetic resonance imaging volumetric data

	Case 1	Case 2	Case 3	Case 4	Case 5	Case 6	*C9ORF72 *	Controls
Age at first scan	63.0	59.2	65.6	70.6	66.8	50.7	62.7 (7.0)	58.7 (5.8)
Gender (male:female)							5:1	10:5
Disease duration (years)	2.9	4.2	7.6	2.6	18.8	2.7	6.5 (6.3)	N/A
Interscan interval (years)	0.9	1.2	1	0.6	1.1	1.1	1.0 (0.2)	1.6 (0.8)
Total intracranial volume (ml)	1,624	1,835	1,854	1,368	1,686	1,465	1,639 (196)	1,610 (144)
Brain volume, baseline (ml)	1,171	1,351	1,290	876	1,232	1,076	1,166 (171)	1,231 (92)
Brain volume, repeat (ml)	1,146	1,344	1,293	842	1,179	1,072	1,146 (179)	1,226 (92)
Volume change (ml)	25	7	-3	34	53	4	20 (22)	5 (17)
Left hemisphere, baseline (ml)	592	675	651	433	623	542	586 (88)	614 (56)
Left hemisphere, repeat (ml)	579	675	653	415	592	538	575 (93)	611 (53)
Right hemisphere, baseline (ml)	589	686	646	451	619	542	589 (84)	612 (60)
Right hemisphere, repeat (ml)	576	681	648	433	595	541	579 (87)	609 (54)
Ventricle volume, baseline (ml)	44.4	38.2	35.0	40.3	27.8	21.8	34.6 (8.4)	21.4 (11.4)
Ventricle volume, repeat (ml)	47.7	39.0	37.9	44.8	29.8	26.2	37.6 (8.3)	22.5 (12.2)
Brain BSI (%/year)	1.1	-0.2	0.5	4.4	1.0	1.6	1.4 (1.6)	0.4 (0.3)
Ventricle expansion (ml/year)	3.6	0.8	2.5	6.2	1.6	4.3	3.2 (2.0)	0.7 (0.6)
Cerebellar BSI (%/year)	-0.5	-0.2	0.6	1.9	1.8	2.6	1.0 (1.3)	0.1 (0.5)
Left hemisphere rate (%/year)	2.4	0.1	-0.4	6.9	4.6	0.6	2.4 (2.9)	0.5 (1.3)
Right hemisphere rate (%/year)	2.4	0.6	-0.2	6.2	3.7	0.1	2.1 (2.5)	0.5 (1.0)

Rates of whole brain, hemispheric and ventricular change. Group data show the mean (standard deviation). BSI, boundary-shift integral; C9ORF72, chromosome 9 open reading frame 72; N/A, not applicable.

### Neuroimaging findings: cortical and subcortical regions

Detailed data on subcortical volume change are displayed in Table 3. Compared with healthy controls, significant subcortical volume loss over time was detected in the *C9ORF72 *mutation group in the right thalamus (*P *= 0.006), left thalamus (*P *= 0.03) and left globus pallidus (*P *= 0.04). No significant change over time was detected in cortical regions when compared with controls.

**Table 3:** Subcortical volumes in *C9ORF72 *mutation and healthy control groups

		*C9ORF72*			Control			
		
		% per year	Atrophy (ml/year)	SD	% per year	Atrophy (ml/year)	SD	*P *value
Thalamus	Right	3.4	0.16	0.17	-0.2	-0.02	0.08	0.006
	Left	2.5	0.23	0.29	-0.3	-0.02	0.09	0.03
Globus pallidus	Right	1.7	0.05	0.10	0.0	0.00	0.03	n/s
	Left	2.9	0.03	0.14	-1.1	-0.02	0.06	0.04
Caudate	Right	-2.6	-0.09	0.15	0.3	0.01	0.06	n/s
	Left	0.7	0.02	0.07	0.0	0.00	0.13	n/s
Amygdala	Right	-2.8	-0.04	0.08	0.1	0.00	0.04	n/s
	Left	2.1	0.03	0.06	0.6	0.01	0.06	n/s
Hippocampus	Right	1.7	0.07	0.08	1.0	0.04	0.06	n/s
	Left	1.4	0.05	0.03	0.6	0.03	0.09	n/s
Putamen	Right	-0.6	-0.03	0.10	0.0	0.00	0.06	n/s
	Left	-1.2	-0.06	0.13	-0.1	0.00	0.10	n/s

Mean annualised rates of change are expressed as a percentage (%) and as the millilitre (ml) change from baseline. C9ORF72, chromosome 9 open reading frame 72; n/s, not significant p < 0.05; SD, standard deviation.

### Nonlinear fluid registrations

**Figure 3:**
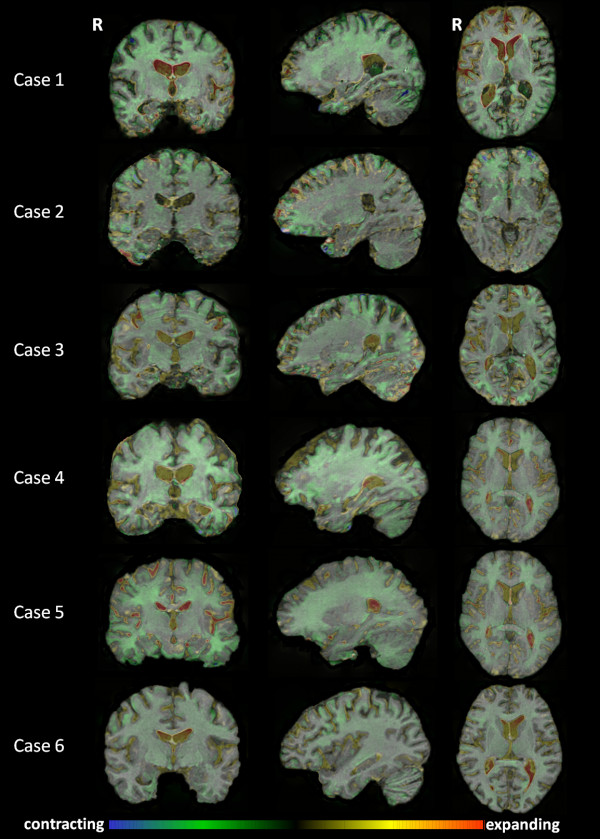
**Coronal, sagittal and axial magnetic resonance imaging images showing areas of contraction and expansion**. Coronal (left), sagittal (middle) and axial (right) magnetic resonance imaging brain slices showing longitudinal voxel-compression maps in individual patients, representing areas of contraction (green-blue) and expansion (yellow-red) over time. Sagittal sections are through the right hemisphere. R, right.

Fluid-based nonrigid registrations in individual patients (Figure 3) revealed heterogeneous patterns of whole brain atrophy across subjects. Over the period of follow-up, most patients showed a diffuse but dorsally directed pattern of cerebral parenchymal loss, with more variable involvement of the temporal lobe regions; ventricular expansion and cerebellar volume loss were consistent features. A pattern of generalised progressive atrophy was apparent in Cases 4 to 6; Case 2 had prominent bifrontal volume loss, particularly implicating orbitofrontal cortices; and Cases 1 and 3 had more posterior atrophy, although expansion of the frontal horns of the lateral ventricles was also prominent in Case 1.

## Discussion and Conclusion

We have described longitudinal neuropsychological and neuroimaging features in a cohort of patients with FTD due to a *C9ORF72 *expansion. Considering the mutation group as a whole, impaired executive function and also episodic memory were early and prominent neuropsychological features. Over follow-up intervals of some 18 months, there was a significant decline in general intellect and a further decline in visual memory, naming and parietal skills, whereas nondominant parietal (visuoperceptual) functions remained relatively intact. Mean brain atrophy and ventricular expansion rates were increased compared with healthy controls and broadly in line with rates of change in previous longitudinal imaging studies of FTD [29,30], although there was substantial diversity across the group. Increased rates of ventricular expansion were consistently observed and may be a candidate biomarker of disease evolution associated with the *C9ORF72 *mutation. Cerebellar atrophy was also a relatively prominent feature in the *C9ORF72 *mutation group, with an approximately 10-fold increase in mean atrophy rate compared with controls. Notably, no specific cortical region appeared disproportionately affected; however, subcortical structures including the thalamus and globus pallidus showed mean rates of atrophy around three times greater than controls. Unlike certain other genetic variants of FTD, notably *GRN *[31], hemispheric atrophy remained largely symmetrical. In further contrast to previous neuroimaging findings in association with mutations of *GRN *(asymmetric fronto-temporo-parietal atrophy) and *MAPT *(antero-medial temporal lobe atrophy) [9], individual atrophy profiles in this *C9ORF72 *mutation cohort were highly variable (Figure 2) - some patients showing chiefly frontal volume loss, whilst others showed relatively more posterior volume loss. Cerebellar atrophy was a relatively consistent feature in individual cases here, although whether this is truly a signature of *C9ORF72*-associated FTD requires substantiation in larger patient cohorts from different centres.

The evolution of cognitive deficits here suggests a distributed disease process implicating frontal, temporal and parietal cortices, particularly in the dominant hemisphere. Degeneration of a distributed subcortical network might reconcile this neuropsychological profile with the rather variable and diffuse profiles of brain atrophy observed here. Degeneration of thalamus, cerebellum and thalamic and frontal white matter tracts has been identified previously in cross-sectional imaging studies of *C9ORF72 *expansions [6,8,9]. In the present study, we provide further evidence that the pathophysiological mechanisms of *C9ORF72-*associated FTD target subcortical networks: rates of thalamic and cerebellar atrophy and ventricular expansion were disproportionately increased relative to whole brain atrophy rates, consistent with involvement of subcortical structures and pathways [32]. The involvement of the globus pallidus observed here is in line with the development of extrapyramidal symptoms in a substantial proportion of *C9ORF72 *cases in other series [33], although our patients did not manifest clear-cut features of Parkinsonism. The thalamus, globus pallidus and cerebellum together act as key hubs coordinating distributed cortico-subcortical circuits and the cognitive operations they mediate [34,35]. Early involvement of such hub regions and projections could facilitate diffusive spread of the molecular pathology responsible for the brain degeneration associated with *C9ORF72 *expansions [36-38] and might be anticipated to lead to rapid clinical evolution, although the very wide range of clinical disease durations among individual patients with a C9ORF72 mutation remains an important unsolved problem. Both the thalamus and cerebellum have been previously implicated in cross-sectional neuroimaging work in *C9ORF72*-associated FTD [6]. The increased prevalence of cerebellar p62 inclusions with *C9ORF72 *expansions compared with other pathologically proven cases of FTD further supports the role of the cerebellum as an important anatomical nidus of *C9ORF72*-associated pathology [6,39].

This study has a number of limitations. Case numbers here were relatively small and individual variation was substantial; larger (ideally, multicentre) longitudinal studies are required to establish the true range of cognitive and neuroimaging features associated with *C9ORF72*-associated FTD and to evaluate candidate biomarkers. The historical nature of the present cohort was a particular limitation on the systematic analysis of behavioural deficits; for example, the nature of the naming impairment here remains ill-defined, and this could in principle reflect primary word retrieval, semantic or mixed deficits. Inclusion of presymptomatic carriers in future studies may allow the earliest behavioural and neuroimaging markers of disease onset to be determined. The specificity of any candidate biomarkers will only be established by comparisons with other genetic and sporadic forms of FTD. We argue that future work should particularly target subcortical (including cerebellar) structures and cognitive functions in the *C9ORF72 *mutation group, incorporating neuroimaging modalities that capture white matter disintegration: although any synthesis must be preliminary, we interpret the present findings as further circumstantial evidence that a distributed cortico-subcortical network is integral to the phenotypic expression of *C9ORF72*-associated FTD.

## Abbreviations

BSI: boundary shift integral; C9ORF72: chromosome 9 open reading frame 72; FTD: frontotemporal dementia; GRN: progranulin; MAPT: micro-tubule protein tau; MRI: magnetic resonance imaging; ORF: open reading frame; PCR: polymerase chain reaction.

## Competing interests

The authors declare that they have no competing interests.

## Authors' contributions

CJM contributed to the conception and design of this study, data collection, data analysis and drafting the manuscript. LED contributed to the data collection and data analysis. GRR contributed to data analysis and drafting of the manuscript. JB contributed to the genetic analysis of subjects. SC, MB, SF, KKL and TY contributed to data analysis. HG contributed to data collection. SM contributed to genetic analysis of subjects. JDR contributed to data collection. NCF contributed to conception and design of the study and review of the manuscript. JDW contributed to the conception and design of this study and drafting and critical revision of the manuscript. All authors read and approved the final manuscript.
